# Functional brain changes in vascular cognitive impairment: a whole brain ALE meta-analysis

**DOI:** 10.3389/fnagi.2025.1521457

**Published:** 2025-06-05

**Authors:** Chunyang Zhang, Mingchen Xue, Han Zhang, Juan Li, Mingli He

**Affiliations:** ^1^Department of Neurology, Lianyungang Clinical College of Nanjing Medical University, Jiangsu, China; ^2^Department of Medical Imaging, Lianyungang Clinical College of Nanjing Medical University, Jiangsu, China

**Keywords:** cognitive impairment, resting state functional magnetic resonance imaging, meta-analysis, amplitude of low-frequency fluctuation, regional homogeneity, functional connectivity

## Abstract

**Background:**

Vascular cognitive impairment (VCI) is a prevalent form of cognitive dysfunction. Resting-state functional magnetic resonance imaging (rs-fMRI) could serve as a potential biomarker for early detection. This study employed activation likelihood estimation (ALE) meta-analysis to investigate specific neural abnormalities in VCI patients.

**Methods:**

We systematically searched PubMed, Embase, and Web of Science for rs-fMRI studies on VCI that reported amplitude of low-frequency fluctuation (ALFF), regional homogeneity (ReHo), or functional connectivity (FC). Sixteen eligible fMRI studies were included in the ALE meta-analysis.

**Results:**

Compared to healthy controls (HCs), VCI patients exhibited the following rs-fMRI alterations. For ALFF, there was an increase in the left anterior cingulate (AC) and left inferior frontal gyrus, possibly a compensatory over - activation. Decreases were seen in regions like the bilateral precuneus and medial frontal gyri (mFG), linked to cognitive deficits. ReHo increased in the left claustrum and insula, suggesting enhanced local synchronization, but decreased in the right sub - gyral region and middle temporal gyru (MTG), which may relate to language issues. FC was enhanced in areas related to complex cognitive processes, yet reduced in regions crucial for memory.

**Conclusion:**

VCI patients exhibited distinct functional abnormalities in specific brain regions, reflecting their diverse cognitive impairments. These region-specific alterations may serve as potential biomarkers for early diagnosis and targeted intervention in VCI.

## Introduction

Vascular cognitive impairment (VCI) encompasses a spectrum of cognitive deficits resulting from cerebrovascular diseases, ranging from mild vascular cognitive impairment (mVCI) and vascular dementia (VaD) (Skrobot et al., 2018; van der Flier et al., 2018). As the second most common cause of dementia, VCI accounts 20%–40% of all diagnoses (Rundek et al., 2022). Early identification and diagnosis of VCI are critically important and have garnered increasing attention. Early identification and diagnosis of VCI are critically important and have garnered increasing attention.

VCI involves complex mechanisms associated both macrostructural and microstructural levels (Badji et al., 2023). Cerebral small vessel disease (CSVD) is widely regarded as the primary driver of VCI pathogenesis, even in the absence of stroke (Inoue et al., 2023; Zanon Zotin et al., 2021). Neuroimaging markers of CSVD include small subcortical infarcts, lacunae, white matter hyperintensities (WMH), enlarged perivascular spaces (EPVS), microbleeds and brain atrophy (Litak et al., 2020). However, the underlying mechanisms linking VCI and CSVD remain highly intricate, posing challenges in identifying consistent pathological patterns across cases.

Disruptions in both structural and functional networks play a key mediating role in how vascular lesions affect cognitive function (Dichgans and Leys, 2017). Resting state functional magnetic resonance imaging (rs-fMRI) leverages spontaneous fluctuations of blood oxygen level dependent (BOLD) signal fluctuations to map functional brain activity, providing a highly reliable and reproducible method for investigating functional connectivity (FC) networks.

Amplitude of low-frequency fluctuation (ALFF), regional homogeneity (ReHo), and FC are widely used rs-fMRI metrics for whole-brain analysis. ALFF measures spontaneous regional neuronal activity, reflecting the brain’s physiological state, while ReHo assesses the synchronization of local neural activity (Jiang and Zuo, 2016; Xi et al., 2012). Higher ALFF values correlate with enhanced cognitive function, indicating increased neuronal excitability (Wang et al., 2020). Conversely, reduced ReHo suggests impaired local neural synchronization, implying abnormal activity in affected brain regions (Wang et al., 2020). FC quantifies temporal correlations in BOLD signals across distinct brain regions or voxels, mapping inter-regional communication (Lin et al., 2018). Together, these metrics provide valuable insights into functional brain variations.

The Vascular Impairment of Cognition Classification Consensus Study (VICCCS) established standardized diagnostic criteria and operational guidelines for VCI (Skrobot et al., 2017; Skrobot et al., 2018). However, the heterogeneous clinical manifestations of CSVD continue to pose significant diagnostic and management challenges (Badji et al., 2023).

Activation likelihood estimation (ALE) is a widely used coordinate-based meta-analysis method that models reported activation foci as spatial probability distributions centered at given coordinates. By computing the union of these probabilities for each voxel, ALE generates statistical maps (thresholded at *p* < 0.05) to identify consistent brain activation patterns across studies (Eickhoff et al., 2009; Turkeltaub et al., 2002). This approach has been extensively applied in rs-fMRI research and holds promise for identifying neuroimaging biomarkers (Xu et al., 2020).

Despite its utility, current VCI research faces several limitations, including small sample sizes, inconsistent inclusion criteria, conflicting findings, and ongoing debate regarding functional network alterations in VCI patients. Although ALE meta-analyses have been conducted in broader cognitive impairment populations, few studies have specifically focused on VCI. Moreover, existing ALE syntheses in this field have primarily examined isolated VCI subtypes (e.g., subcortical vascular cognitive impairment or vascular mild cognitive impairment), leaving a critical gap in comprehensive, spectrum-wide analyses (Xu et al., 2021; Zhang X. et al., 2021).

To address this, our study aimed to perform a systematic ALE meta-analysis of VCI, with three key objectives: (1) Identify rs-fMRI differences between VCI patients and healthy controls (HCs) to assess its diagnostic biomarker potential;(2) Investigate the relationship between altered brain regions and cognitive deficits in VCI;(3) Provide an integrative synthesis of functional network disruptions across the VCI spectrum.

## Methods

The meta-analysis of neuroimaging studies was conducted according to the Preferred Reporting Items for Systematic Reviews and Meta-Analyses (PRISMA) statement and recorded using the PRISMA 2020 Checklist (Page et al., 2021).

### Literature search and article selection

#### Database search

A systematic literature search was conducted in PubMed, Web of Science, and Embase (accessed on 13 September 2024) using the following key terms: VCI-related terms:(“vascular cognitive impairment” OR “vascular dementia” OR “vascular cognitive disorder” OR “VCI” OR “VD” OR “VaD”). Neuroimaging markers: AND (“amplitude of low-frequency fluctuation” OR “ALFF” OR “regional homogeneity” OR “ReHo” OR “functional connectivity” OR “FC”). Given the broad spectrum of VCI, additional terms were included to capture relevant subtypes and etiologies:(“small vessel disease” OR “vascular cognitive impairment-no dementia” OR “vascular cognitive impairment not dementia” OR “subcortical ischemic vascular disease” OR “recent small subcortical infarct” OR “white matter hyperintensity” OR “cerebral microbleed” OR “Leukoaraiosis” OR “leukodystrophy” OR “CADASIL”). Searches were limited to English-language publications.

#### Literature screening process

**FIGURE 1:**
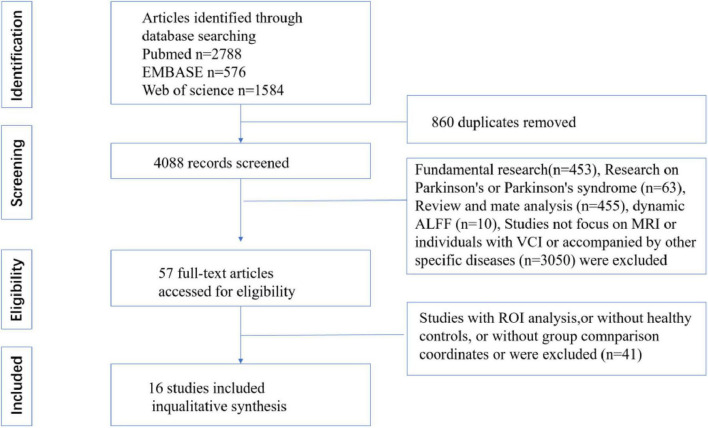
Flowchart shows study selection process.

Two independent researchers conducted the literature search and screening. Discrepancies were resolved through discussion with a third reviewer. The screening process consisted of four sequential steps: (1) Title/Abstract Screening: Initial exclusion of irrelevant studies; (2) Full-Text Review: Further assessment of potentially eligible articles; (3) Final Eligibility Check: Detailed evaluation of remaining studies; (4) Cross-Verification: Ensured no relevant studies were omitted. After this rigorous selection process, 16 articles met the inclusion criteria. The PRISMA flow diagram (Figure 1) illustrates the search and screening process.

#### Inclusion criteria

• Studies were included if they met all of the following criteria:
• Study Design: Resting-state fMRI (rs-fMRI) investigations of VCI.
• Participants: Included both VCI patients and HCs, with baseline data comparisons between groups.
• Analysis Method: Whole-brain analysis (not restricted to ROI-based approaches).
• Reporting Standards: Provided Talairach or MNI coordinates for group-level comparisons (VCI vs. HCs).
• Outcome Measures: Reported differences in ALFFs, ReHo, or FC.

#### Exclusion criteria

Studies were excluded if they met any of the following conditions:

• Publication Type: Reviews, meta-analyses, case reports, animal studies, letters, protocols, theoretical models, conference abstracts, commentaries, or books.
• Patient Population: Included individuals with Parkinson’s disease, Alzheimer’s disease (AD), frontotemporal dementia, psychiatric disorders, or acute cerebral infarction/intracerebral hemorrhage with a disease duration of less than 6 months.
• Etiological Confounders: Enrolled patients with leukoencephalopathy due to immune, toxic, metabolic, or neoplastic causes.
• Unclear Classification: Studies that could not be definitively classified as VCI-related research.

### Quality assessment and data extraction

Given the lack of standardized tools for evaluating methodological quality in fMRI meta-analyses, we adapted the Newcastle-Ottawa Scale (mNOS) (Costa et al., 2021; Gentili et al., 2019; Wells et al., 2000) to assess study quality and risk of bias. The mNOS scoring system (range: 0–11) categorized studies as: High risk (0–3), Intermediate risk (4–7), and Low risk (8–11) (Costa et al., 2021). Two independent researchers performed the assessments, with inter-rater reliability evaluated using Cohen’s Kappa statistic. Discrepancies were resolved through consensus or consultation with a third reviewer.

#### Data extraction

The following variables were systematically extracted: (1) Demographics: Sample size, sex distribution, mean age; (2) Cognitive measures: Mini-Mental State Examination (MMSE), Montreal Cognitive Assessment (MoCA); (3) Imaging data: Group contrasts, peak coordinates (focus), significance thresholds (*p*-values); (4) Subgroup classification (if applicable).

### Meta-analysis procedures

Spatial convergence analysis was performed using GingerALE 3.0.2^1^ (Eickhoff et al., 2009; Eickhoff et al., 2012; Turkeltaub et al., 2012). All included studies reported coordinates in MNI space, eliminating the need for spatial normalization.

The ALE algorithm models each reported focus as a 3D Gaussian probability distribution (accounting for spatial uncertainty), then computes the union of these distributions across studies to identify statistically convergent activation patterns. This approach inherently controls for methodological heterogeneity (e.g., varying preprocessing pipelines, statistical thresholds, or cohort characteristics) by: (1) Weighting voxels based on cross-study consistency; (2) Applying cluster-level inference (primary threshold: *p* < 0.05, FWE-corrected via 1000 permutations. Resulting ALE maps were visualized on the MNI152 template using Mango V4.1^2^, with anatomical labeling according to the AAL3 atlas.

## Results

### Research results

Following literature screening, 16 studies were included in the meta-analysis. VCI patients were categorized into the following subgroups based on diagnostic criteria: “VCI”, “vascular mild cognitive impairment (VaMCI)”, “vascular cognitive impairment, no dementia (VCIND)”, “CSVD with mild cognitive impairment (CSVD-M)”, “pontine stroke (PS)”, “subcortical ischemic vascular dementia (SIVD)”, “subcortical ischemic vascular disease with CI (SIVD-CI)”, “subcortical ischemic vascular disease with mild cognitive impairment (SIVD-MCI)”, “subcortical ischemic vascular disease with cognitive impairment (SIVD-CI)”, “CSVD with cognitive impairment (CSVD-CI)”, “white matter hyperintensities with CI (WMH-CI)”, “leukoaraiosis with vascular mild-cognitive impairment (LA-VaMCI)”, “leukoaraiosis with vascular-dementia (LA-VaD)”. For the ALFF analysis, 8 studies (involving 508 participants: 256 VCI patients and 252 HCs) revealed that VCI patients had increased ALFF in 20 foci and decreased ALFF in 20 foci compared to HCs. The ReHo analysis included 6 studies (316 participants: 149 VCI patients, 167 HCs), showing increased ReHo in 8 foci and decreased ReHo in 15 foci in VCI patients versus HCs. The FC analysis, comprising 4 studies (254 participants: 121 VCI patients, 133 HCs), demonstrated enhanced FC in 5 foci and reduced FC in 13 foci in VCI patients relative to HCs, with detailed data presented in Table 1.

**TABLE 1:** Demographic data and clinical information.

Study	Group	*N*	Age	Sex (male/ female)	MMSE	MocA	Group contrasts	Foci	Correction for multiple comparisons
**ALFF**									
Li et al., 2021b	VaMCI	31	62.87 ± 7.07	20-Nov	–	23 (20, 24)	–	–	–
	HCs	31	59.35 ± 8.15	14/17	–	28 (26, 30)	VaMCI < HC	4	*p* < 0.05 (cor)
Ding et al., 2018	VCIND	14	67.9 ± 8.7	08-Jun	26.87 ± 0.32	20.32 ± 3.72	VCIND < HC	2	*p* < 0.05 (cor)
	HCs	15	65.8 ± 7.9	07-Aug	28.51 ± 0.28	26.33 ± 2.98	VCIND < HC	7	*p* < 0.05 (cor)
Zhang et al., 2023	CSVD-M	19	67.89 ± 8.01	10-Sep	26.63 ± 1.61	23.47 ± 2.01	CSVD-M < HC	1	*p* < 0.05 (cor)
	HCs	18	61.67 ± 7.62	06-Dec	27.71 ± 1.57	26.53 ± 0.62	CSVD-M < HC	2	*p* < 0.05 (cor)
Liu et al., 2014	SIVD	30	69.0 ± 7.8	19-Nov	16.1 ± 5.1	9.4 ± 3.8	SIVD < HC	3	*p* < 0.01 (cor)
	HCs	35	68.0 ± 5.8	22/13	28.4 ± 1.1	27.2 ± 1.5	SIVD < HC	1	*p* < 0.01 (cor)
Song Z. et al., 2023	SIVD-CI	32	75.09 ± 8.68	15/17	20.91 ± 4.19	17.91 ± 4.50	SIVD-CI < HC	1	*p* < 0.05 (cor)
	HCs	32	73.36 ± 7.26	17/15	28.00 ± 2.32	27.61 ± 1.86	SIVD-CI < HC	1	*p* < 0.05 (cor)
Zhang X. et al., 2021	VaMCI	32	69.54 ± 7.23	18/14	24.11 ± 1.01	20.78 ± 1.52	VaMCI < HC	5	*p* < 0.05
	HCs	30	65.3 ± 9.38	17/13	27.46 ± 1.23	27.01 ± 1.12	–	–	–
Song J. et al., 2023	CSVD-CI	52	69.63 ± 5.75	31/21	22.58 ± 4.19	–	CSVD-CI < HC	7	*p* < 0.001 (cor)
	HCs	63	67.62 ± 5.56	30/32	27.92 ± 1.56	–	CSVD-CI < HC	4	*p* < 0.001 (cor)
Wang et al., 2019	LA-VaMCI	28	59.28 ± 6.12	14/14	24.96 ± 1.48	21.68 ± 2.74	LA-CI < HC	1	*p* < 0.05 (cor)
	LA-VaD	18	60.28 ± 11.65	08-Oct	20.53 ± 1.77	17.17 ± 2.09			
	HCs	28	58.35 ± 6.82	13/15	29.46 ± 1.07	28.64 ± 1.66	LA-CI < HC	1	*p* < 0.05 (cor)
**ReHO**									
Zuo et al., 2018	VaMCI	31	63.84 ± 14.1	18/13	26.32 ± 2.06	23.32 ± 1.33	–	–	–
	HCs	32	62.72 ± 8.22	18/14	26.32 ± 2.06	27.75 ± 1.72	VaMCI < HC	2	*p* < 0.05 (cor)
Liu et al., 2021	SIVD-MCI	28	70.73 ± 5.58	16-Dec	23.93 ± 1.90	–	–		*p* < 0.05 (cor)
	HCs	24	68.43 ± 8.02	Oct-14	28.00 ± 1.06	–	SIVD-MCI < HC	3	*p* < 0.05 (cor)
Zhang X. et al., 2021	VaMCI	32	69.54 ± 7.23	18/14	24.11 ± 1.01	20.78 ± 1.52	VaMCI < HC	4	*p* < 0.05
	HCs	30	65.3 ± 9.38	17/13	27.46 ± 1.23	27.01 ± 1.12	VaMCI < HC	3	*p* < 0.05
Tu et al., 2020	SIVD	20	75.8 ± 7.67	13-Jul	–	–	SIVD < HC	–	–
	HCs	23	65.1 ± 6.97	11-Dec	–	–	SIVD < HC	1	*p* < 0.001
Ye et al., 2019	WMH-CI	14	66.00 ± 5.13	07-Jul	26.86 ± 2.66	20.43 ± 2.71	WMH-CI < HC	2	*p* < 0.001
	HCs	33	62.03 ± 7.53	16/17	28.47 ± 1.49	26.41 ± 2.30	WMH-CI < HC	1	*p* < 0.001
Cai et al., 2023	VCI	24	63.75 ± 4.27	Nov-13	21.12 ± 0.33	20.37 ± 1.24	VCI < HC	2	*p* < 0.05 (cor)
	HCs	25	60.60 ± 3.95	Dec-13	29.76 ± 0.43	29.00 ± 0.64	VCI < HC	5	*p* < 0.05 (cor)
**FC**									
Ding et al., 2018	VCIND	14	67.9 ± 8.7	08-Jun	26.87 ± 0.32	20.32 ± 3.72	VCIND < HC	4	*p* < 0.05 (cor)
	HCs	15	65.8 ± 7.9	07-Aug	28.51 ± 0.28	26.33 ± 2.98	VCIND < HC	4	*p* < 0.05 (cor)
Wang et al., 2022	PS	47	57.75 ± 7.40	26/21	–	–	–	–	–
	HCs	55	55.77 ± 8.03	33/23	–	–	PS < HC	8	*p* < 0.01
Liu et al., 2019	SVCI	29	70.48 ± 5.76	16/13	24.00 ± 1.91	–	SVCI < NC	1	*p* < 0.05 (cor)
	HCs	27	67.63 ± 8.19	Oct-17	27.93 ± 1.03	–	–	–	–
Li et al., 2021a	VaMCI	31	64.93 ± 10.11	18/13	–	23.32 ± 1.32	–	–	
	HCs	36	64.22 ± 6.97	17/19	–	25.22 ± 2.89	VaMCI < HC	1	*p* < 0.05 (cor)

VaMCI, vascular mild cognitive impairment; HCs, healthy controls; VCIND, vascular cognitive impairment, no dementia; CSVD-M, CSVD with mild cognitive impairment; SIVD, subcortical ischemic vascular dementia; SIVD-CI, subcortical ischemic vascular disease with cognitive impairment; CSVD-CI, CSVD with cognitive impairment; LA-VaMCI, leukoaraiosis with vascular mild-cognitive impairment; LA-VaD, leukoaraiosis with vascular-dementia; SIVD-MCI, subcortical ischemic vascular disease with mild cognitive impairment; WMH-CI, white matter hyperintensities with cognitive impairment; PS, pontine stroke.

### Meta-analysis results

#### Altered ALFF in VCI patients

**FIGURE 2:**
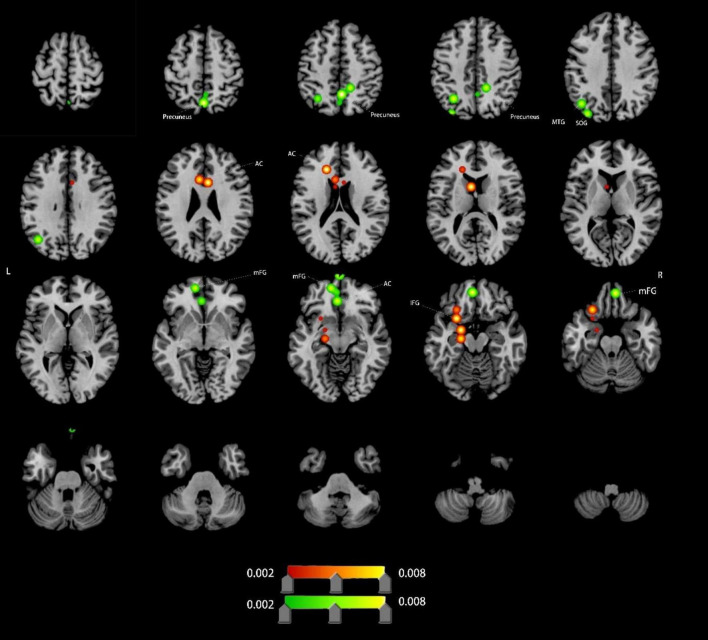
Brain regions showing increased/decreassed ALFF in VCI patients compared to HCs. Red indicates regions of increased ALFF values. Green indicates regions of decreased ALFF values. AC, anterior cingulate; IFG, inferior frontal gyrus; mFG, medial frontal gyrus; MTG, middle temporal gyrus; SOG, superior occipital gyrus.

Compared to HCs, VCI patients demonstrated significant ALFF alterations, characterized by increased ALFF (4 clusters), left anterior cingulate cortex (ACC), left inferior frontal gyrus (IFG), decreased ALFF (11 clusters),left ACC (distinct subregion from increased cluster), bilateral medial frontal gyrus (mFG), bilateral precuneus, left middle temporal gyrus (MTG) and left superior occipital gyrus (SOG). Detailed spatial distributions and statistical thresholds are presented in Figure 2 and Table 1.

#### Altered ReHo in VCI patients

**FIGURE 3:**
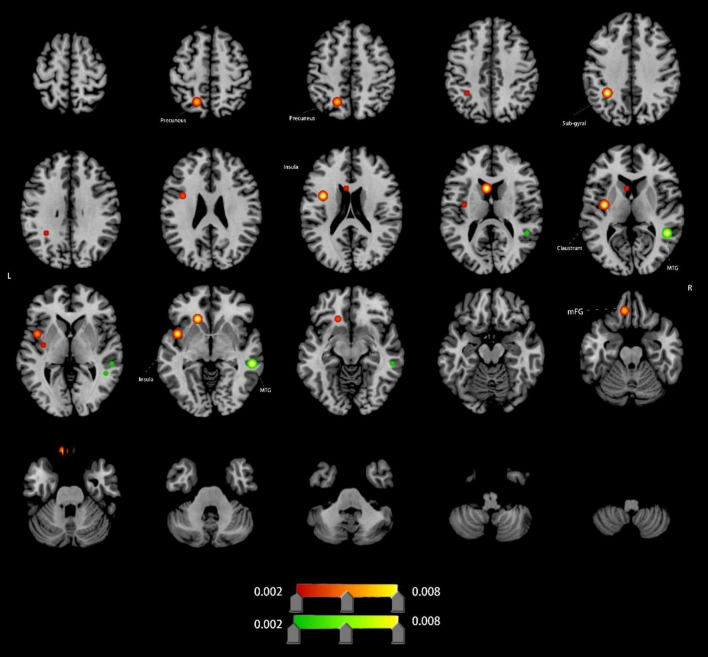
Brain regions showing increased/decreassed ReHo in VCI patients compared to HCs. Red indicates regions of increased ReHo values. Green indicates regions of decreased ReHo values. MTG, middle temporal gyrus; mFG, medial frontal gyrus;

Compared to HCs, VCI patients exhibited significant ReHo differences. Increased ReHo (8 clusters): left claustrum, left insula, left sub-gyral region, left mFG and left precuneus; Decreased ReHo (2 clusters): right sub-gyral and right MTG (Figure 3 and Table 1).

#### Altered FC in VCI patients

**FIGURE 4:**
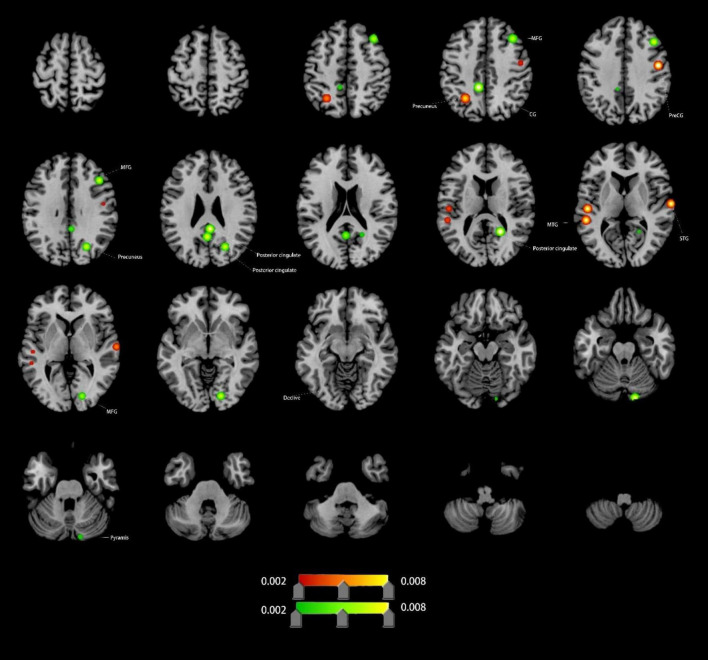
Brain regions showing increased/decreassed FC in VCI patients compared to HCs. Red indicates regions of increased FC values. Green indicates regions of decreased FC values. PreCG, precentral gyrus; MFG, middle frontal gyrus; MTG, middle temporal gyrus; STG, superior temporal gyrus; CG, cingulate gyrus; LING, lingual gyrus.

Compared to HCs, VCI patients exhibited significant FC differences. Increased FC (4 clusters): right precentral gyrus (PreCG), left MTG, left precuneus, right superior temporal gyrus (STG); Decreased FC (9 clusters): left cingulate gyrus (CG), both posterior cingulate, right precuneus, right lingual gyrus (LING), right pyramis, right PreCG, and right MFG (Figure 4 and Table 1).

## Discussion

This study investigated resting-state network alterations in VCI patients compared to HCs, focusing on amplitude of ALFF, ReHo, and FC. These metrics capture distinct yet complementary aspects of neural activity (ALFF), local synchronization (ReHo), and inter-regional communication (FC), offering a comprehensive view of VCI-related dysfunction. By identifying aberrant functional signatures, our findings may contribute to the development of probabilistic biomarkers for early VCI detection, facilitating timely and targeted interventions.

**ALFF Abnormalities:** ALFF alterations were predominantly localized in prefrontal, precuneus, and temporal regions. Increased ALFF in the left AC and IFG suggests enhanced neuronal excitability, potentially reflecting compensatory mechanisms for cognitive control, emotional processing, and multitasking in response to cerebral ischemia (Briggs et al., 2019; Kolling et al., 2016; Monosov et al., 2020; Sato et al., 2023; Shinozaki et al., 2016; Shu et al., 2022). Conversely, decreased ALFF in mFG, bilateral precuneus, and other default mode network (DMN) hubs indicates resting-state dysfunction, likely contributing to episodic memory decline, executive dysfunction, and emotional dysregulation in VCI (Dadario and Sughrue, 2023; Frascarelli et al., 2015; Myung et al., 2016).

**ReHo Alterations:** ReHo analysis revealed increased local synchronization in the left claustrum and mFG, suggesting adaptive changes in decision-making and executive control networks. In contrast, decreased ReHo in the right MTG implies disrupted language network coordination, aligning with semantic deficits in VCI (Briggs et al., 2021; Cui et al., 2020; Frascarelli et al., 2015; Jackson et al., 2020; Madden et al., 2022).

**FC Changes:** Enhanced FC in the right STG, MTG, and left precuneus may reflect enhancements in speech perception, auditory word comprehension, and language processing, as complex cognitive processes that require neural integration across multiple brain regions (Bhaya-Grossman and Chang, 2022; Dadario and Sughrue, 2023; Liu et al., 2023; Sugimoto et al., 2023). Conversely, reduced FC in the cingulate gyrus (CG), posterior cingulate, and precuneus reflects DMN disintegration, correlating with impaired self-referential processing and memory consolidation (Bubb et al., 2018; Dadario and Sughrue, 2023; Foster et al., 2023; Leech and Sharp, 2014).

The findings strongly support CSVD as the principal pathological basis of VCI. CSVD-related structural damage—including WMH and lacunar infarcts—likely disrupts critical neural pathways, leading to widespread functional network dysfunction (Chen et al., 2018; Zanon Zotin et al., 2021). This disorganization particularly affects hub regions such as the precuneus, a key node in the default mode network (DMN) that is highly vulnerable to hypoperfusion in CSVD (Dadario and Sughrue, 2023; Love and Miners, 2016). In VCI patients, reduced ALFF and FC in the precuneus may underlie diverse cognitive deficits, particularly in spatial processing and navigation (Cavanna and Trimble, 2006), manifesting clinically as disorientation and impaired spatial cognition. Notably, our meta-analysis—encompassing multiple VCI subtypes (VaMCI, VaD, CSVD-related cognitive impairment) —revealed consistent functional abnormalities across the VCI spectrum, aligning with VICCCS guidelines that advocate for multidimensional neuroimaging markers in VCI diagnosis (Skrobot et al., 2017; Skrobot et al., 2018). By integrating ALFF, ReHo, and FC across the VCI continuum, this study provides preliminary evidence for their utility as complementary diagnostic biomarkers, potentially enhancing early detection and stratification of VCI.

Multimodal neuroimaging reveals a dynamic inter play in VCI, characterized by regional hyperactivity (↑ALFF/ReHo) alongside network disconnection (↓FC) —reflecting concurrent neuroplastic adaptation and pathological decompensation (Fornito et al., 2015). While the brain exhibits compensatory mechanisms to preserve homeostasis, these processes are complex and multifaceted, with VCI patients demonstrating more pronounced structural and functional alterations than typical aging (Jin et al., 2025).

Current limitations—such as heterogeneous datasets and inconsistent VCI subtyping—highlight the need for large-scale, prospective studies integrating multimodal neuroimaging (rs-fMRI, DTI, structural MRI) with detailed clinical profiles. Such efforts should: (1) Validate subtype-specific abnormalities across diverse VCI cohorts (e.g., VaMCI, VaD, CSVD-related cognitive impairment); (2) Clarify mechanistic links between functional disruptions (ALFF/ReHo/FC) and structural/metabolic changes; (3) Build upon this meta-analysis as an exploratory foundation for personalized diagnostic and therapeutic strategies.

This study has several limitations that warrant consideration. First, heterogeneity in meta-analysis, despite strict inclusion/exclusion criteria, variability in data sources, preprocessing methods, statistical thresholds, and imaging protocols was unavoidable, potentially influencing the results. Second, in whole-Brain Approach vs. Network-Specific Focus, the whole-brain ALE analysis—rather than targeting specific networks—limited the depth of investigation and may have excluded relevant studies. Third, subtype analysis challenges, the broad and heterogeneous nature of VCI, combined with the limited number of eligible studies, precluded meaningful subgroup analyses (e.g., VaMCI vs. VaD). Finally, in ALE methodological constraints, the ALE technique lacks significance testing for individual contributing studies, restricting quantitative interpretation of regional findings.

## Conclusion

This ALE meta-analysis identified consistent rs-fMRI abnormalities (ALFF/ReHo/FC) in key cognitive hubs—including the cingulate gyrus (CG), precuneus, and anterior cingulate (AC)—providing mechanistic insights into VCI-related functional impairments. These disruptions may serve as early diagnostic biomarkers, enabling targeted interventions for at-risk patients before overt cognitive decline manifests.

## Data Availability

The raw data supporting the conclusions of this article will be made available by the authors, without undue reservation.

## Footnotes

1
http://www.brainmap.org/ale

2
https://mangoviewer.com/mango
